# Brexucabtagene autoleucel for relapsed/refractory mantle cell lymphoma in asia: redefining the role of allogeneic haematopoietic stem cell transplantation

**DOI:** 10.1007/s00277-026-07126-6

**Published:** 2026-06-19

**Authors:** Lawrence Cheng Kiat Ng, Jing Yuan Tan, Shin Yeu Ong, Chandramouli Nagarajan, Michelle Li Mei Poon, Aloysius Yew Leng Ho, Francesca Wei Inng Lim, Yunxin Chen

**Affiliations:** 1 Department of Haematology, Singapore General Hospital, Singapore, Singapore; 2 Duke-NUS Medical School, Singapore, Singapore; 3 Department of Haematology-Oncology, National University Cancer Institute, National University Hospital, Singapore, Singapore; 4 Yong Loo Lin School of Medicine, Singapore, Singapore

**Keywords:** Brexu-cel, AlloHSCT, Mantle cell lymphoma, Asia

## Abstract

Brexucabtagene autoleucel (brexu-cel) is an autologous CD19-directed CAR T-cell therapy approved for relapsed/refractory (R/R) mantle cell lymphoma (MCL) based on the pilot study ZUMA-2. Since then, several US and European real-world studies have demonstrated comparable efficacy outcomes. However, these reports have also suggested potentially higher rates of acute toxicities, particularly immune effector cell–associated neurotoxicity syndrome (ICANS), raising concerns regarding its use, especially in vulnerable populations. At Singapore General Hospital, five patients (median age 75 years) received brexu-cel from April 2025 to Dec 2025 for R/R MCL. Historical cohort MCL patients received allogeneic haematopoietic stem cell transplantation (allo-HSCT) were extracted and compared. For brexu-cel cohort, the overall response rate (ORR) was 100%, with a complete response rate (CRR) of 80%. Cytokine release syndrome (CRS) occurred in 80% of patients, with no grade 3 events, and only one patient developed grade 1 ICANS (20%). These efficacy and safety outcomes compare favourably with historical cohorts of patients who underwent allo-HSCT locally (total 6 patients, 2 relapsed, 2 NRM), despite potentially higher-risk features and less controlled disease at baseline. Efficacy and toxicity of both clinical trial and real-world CAR-T studies for R/R MCL were summarized. To our knowledge, this represents the first real-world report from Asia on brexu-cel in R/R MCL, supporting its use in this region, where published data have thus far predominantly originated from the United States and Europe.

## Introduction

Mantle cell lymphoma (MCL) is an uncommon yet aggressive subtype of B-cell non-Hodgkin lymphoma characterized by relapse and diminishing responses to successive lines of therapy [1, 2]. Although the introduction of Bruton tyrosine kinase inhibitors (BTKi) has significantly improved outcomes in the relapsed/refractory (R/R) setting, prognosis remains poor following BTKi failure, with median overall survival historically reported between 3 and 11 months [3, 4], underscoring a critical unmet need for effective salvage strategies.

Historically, allogeneic hematopoietic stem cell transplant (alloHSCT) was the only curative option for R/R MCL [5]. However, due to the significant toxicities and non-relapse mortality (NRM), only younger and fitter patient could potentially benefit from it. Moreover, disease remission status also plays an essential deciding factor for whoever eligible for alloHSCT, further restricting the eligibility for those patients who can undergo this therapy.

Lately, cellular immunotherapies have reshaped the treatment landscape of B-cell lymphoma including R/R MCL. Brexucabtagene autoleucel (brexu-cel), an autologous CD19-directed chimeric antigen receptor (CAR) T-cell therapy, produced deep and durable remissions with a median overall survival exceeding 46 months [6, 7], while real-world analyses have demonstrated response rates above 90% [8–10], supporting the reproducibility of these outcomes outside clinical trials. Another CD19 CAR-T, Lisocabtagene Maraleucel (Liso-cel) also showed impressive result in R/R MCL [11]. These results led to FDA and EMA approvals.

In Asia, the only report of CAR-T cell therapy in R/R MCL came from Relmacabtagene autoleucel in China [12]. Seventy patients were enrolled in the phase 2 study, with 59 patients received the infusion showed promising efficacy and manageable toxicities. Unfortunately, this treatment is only available in China. Hence, there is limited real world data describing the use of CAR-T therapy in R/R MCL in Asia.

In Singapore, Brexu-cel was approved for R/R MCL after at least two prior lines of therapies since Aug 2024. Here, we describe the experience of a Singapore tertiary center, reporting consecutive patients with R/R MCL treated with brexu-cel alongside historical cohort of R/R MCL patients underwent alloHSCT.

## Methods

### Study design and patients

From the transplant and cell therapy registry database of Singapore General Hospital, (CRIB 2015–2419) retrospective analysis was performed on consecutive patients diagnosed with R/R MCL who received Brexu-cel from April 2025 to Dec 2025. All patients had the diagnosis of MCL histologically confirmed by local haematopathologist accordance with the World Health Organization classification system [13, 14]. The institutional review board approved the protocols and analyses, and informed consent was obtained from all patients included in the registry. Standard definitions were used to determine disease stage and response.

## Brexu-cel cohort

Patients in the Brexu-cel cohort underwent leukapheresis followed by infusion of autologous anti-CD19 CAR-T cells after lymphodepleting chemotherapy with fludarabine and cyclophosphamide. Supportive care and toxicity management followed institutional guidelines, guided by international guideline [15]. In view of high risk of fungal infection, antifungal prophylaxis was given during neutropenia period [16, 17]. 

## Historical cohort of alloHSCT

Data of consecutive MCL patients underwent alloHSCT from two transplant centers in Singapore (SGH/NUH) reported on a previous publication were extracted from existing database [18]. As per the publication, donor selection, conditioning chemotherapy, GVHD prophylaxis were by transplant physician discretion according to local institution protocol, guided by international guideline [19]. 

## Definition of response and survival outcomes

Date of last follow up was 31 January 2026. For patient underwent Brexu-cel, disease status and response assessment was done with PET-CT scan as per Lugano criteria [20]. CRS and ICANS were graded using ASTCT criteria [21]. 

## Results

### Patient characteristics

There were 5 R/R MCL patients included in this study. Median age was 75 years (65–76), with 3 females and 2 males. 3 out of 5 had blastoid morphology with high ki-67 above 50%. Two patients had documented tp53 mutation disease. The median lines of prior therapies were 4 (2–4) with all of them refractory to covalent BTK-inhibitor. Three had resistance disease to venetoclax and one received bispecific antibody. All patients received systemic bridging, with three patients received radiotherapy bridging. At CAR-T infusion, all patients were in stable disease/progressive disease (SD/PD), while all but one had elevated LDH. Table 1.

**Table 1:** Characteristics and Outcomes of Patients underwent Brexu-cel

	Patient 1	Patient 2	Patient 3	Patient 4	Patient 5
Age/Gender	75/F	69/F	65/F	75/M	76/M
MIPI at diagnosis	Intermediate	High	High	High	High
Ki-67 (%)	20	80	60	70	35
TP53 mutation	Positive	Not done	Not done	Positive	Negative
Morphology	Classical	Blastoid	Blastoid	Blastoid	Classical
Line of prior systemic therapies	4	4	4 (including alloHSCT)	3	2
First Line regimen	BR	NORDIC	NORDIC	Acalabrutinib-BR	Acalabrutinib-BR
Maintenance Rituximab	No	Yes	No	No	Yes
Second Line regimen	RCHOP	Ibrutinib	Acalabrutinib	Venetoclax-Cytarabine	Pirtobrutinib
Third Line regimen	Ibrutinib-Lenalidomide	RBAC	Venetoclax-AlloHSCT	Glofitamab-lenalidomide	
Fourth Line regimen	Clinical Trial (AntiROR1)	Venetoclax	Acalabrutinib-Venetoclax		
Bridging therapy	Systemic + RT	Systemic + RT	Systemic + RT	Systemic	Systemic
Disease status at CAR-T infusion	SD	PD	SD	PD	SD
LDH at CAR-T infusion	Normal	Elevated	Elevated	Elevated	Elevated
CRS grade	1	1	2	1	0
Onset of CRS	D3	D3	D7	D8	None
Doses of Tocilizumab	3	1	2	0	0
ICANS grade	1	None	None	None	None
Onset of ICANS	D9	NA	NA	NA	NA
Cumulative dose of dexamethasone	320 mg	0	0	10 mg	0
Cumulative dose of anakinra	1700 mg	0	0	0	0
Infection grade	Grade 3	None	None	None	None
Follow up duration (day)	265	286	253	195	31
Month-1 response assessment	CR	CR	CR	CR	PR
Month-3 response assessment	CR	CR	CR	CR	Pending
Clinical remission at 6 months	Yes	Yes	Yes	Yes	Pending

Brexu-cel, brexucabtagene autoleucel; alloHSCT, allogeneic haematopoetic stem cell transplant; BR, bendamustine-rituximab; RBAC, rituximab-bendamustine-cytarabine; PD, progressive disease; CAR-T, chimeric antigen receptor t-cell; RT, radiotherapy; LDH, lactate dehydrogenase; CRS, cytokine release syndrome; ICANS, immune-effector cell associated neurotoxicity syndrome; CR, complete response

## Treatment efficacy and survival outcomes

**Fig. 1:**
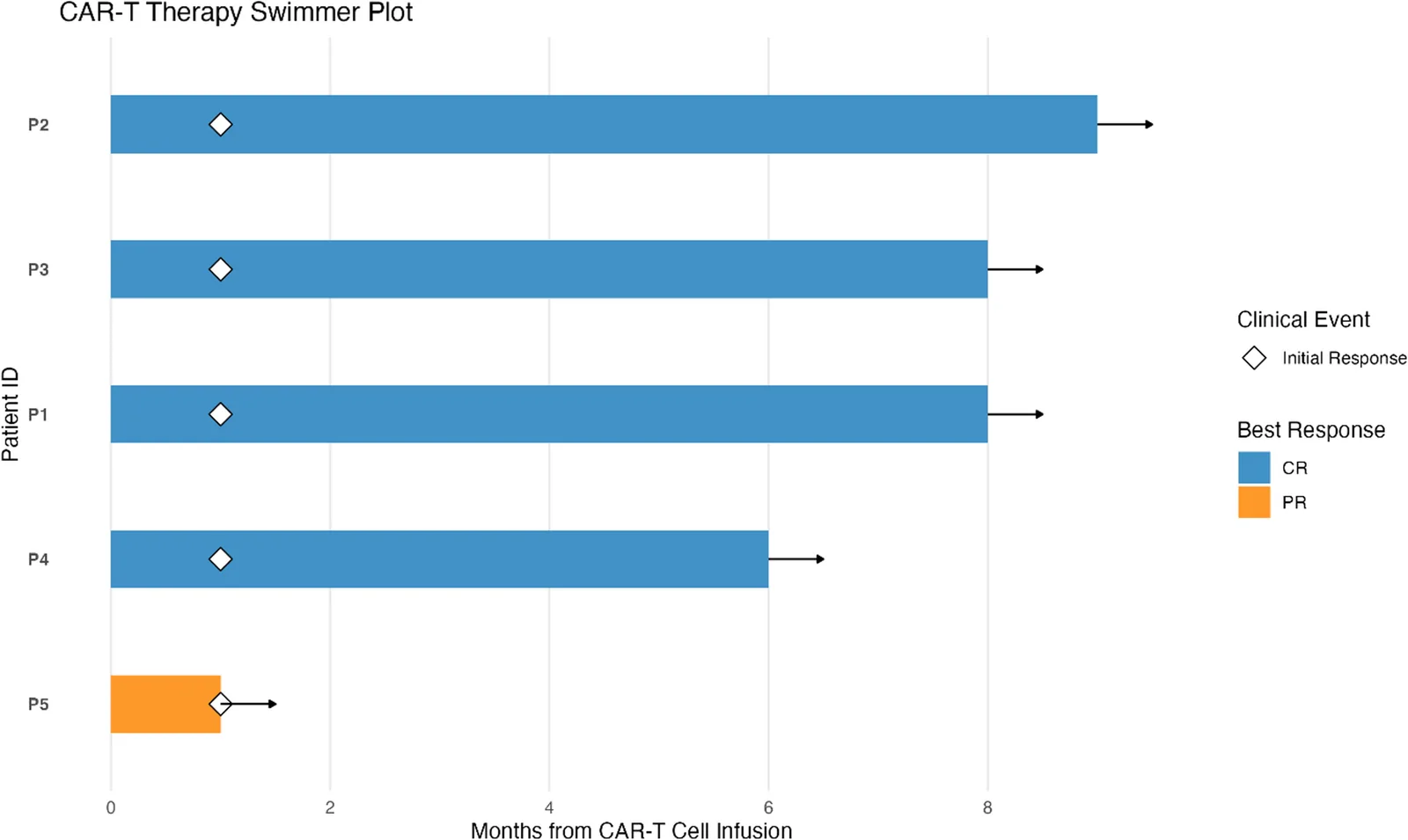
Swimmer plot for patients underwent brexu-cel (*n* = 5)

In the CAR-T cohort, the overall response rate was 100%, with complete remission (CR) achieved in 80%. With the 4 patients who had follow-up duration more than 6-months (195–286 days), all had remained in CR and alive. One patient who was 31 days post CAR-T, were in PR and alive. Figure 1. **Safety and Toxicity**.

CRS occurred in 80% of CAR-T–treated patients (three grade 1 and one grade 2), with no cases of grade ≥ 3 CRS. The onset of CRS ranged from day 3 to day 8. Tocilizumab was administered in three cases, with one, two, and three doses given, respectively.

ICANS was observed in only one patient (grade 1), with onset on day 9. This patient received a cumulative dose of 320 mg of dexamethasone and 1,700 mg of anakinra due to concomitant steroid-induced altered mental status, which complicated the management of ICANS. The neurologic symptoms resolved completely.

Two patient developed grade 3 infection (one with fungal lung infection, one with rhinovirus pneumonia). Both recovered fully. Two patients with hypogammaglobulinemia received regular intravenous immunoglobulin. No secondary malignancies have been observed to date during the follow-up period. Table 1.

## AlloHSCT cohort

**Fig. 2:**
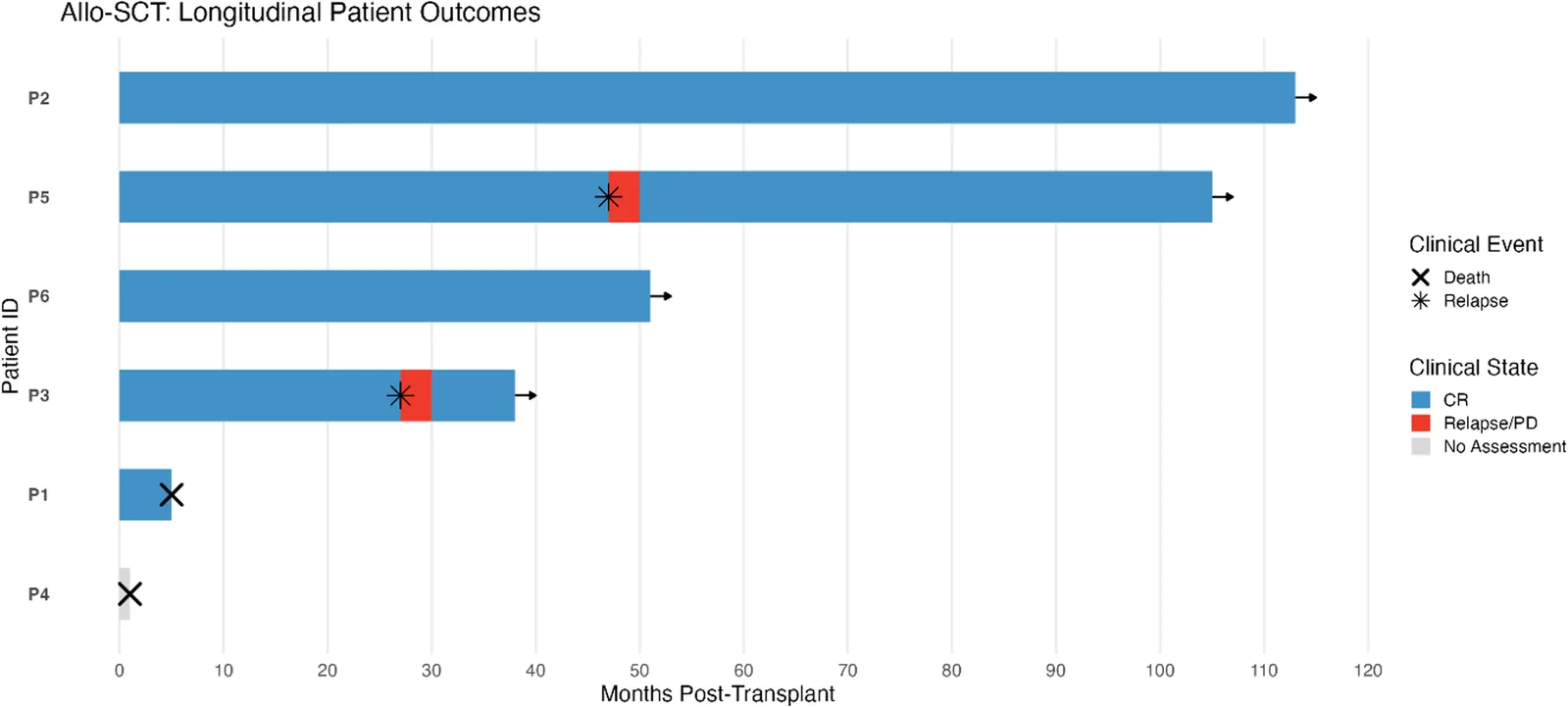
Swimmer plot for historical patients underwent alloHSCT (*n* = 6)

Historical cohort of patients who received alloHSCT at two major transplant centers in Singapore (SGH/NUH) from 2010 to 2024 (pre-Brexu-cel era) were examined. There were 6 patients, median age 51 years. Three patients with prior autologous stem cell transplant (ASCT). 5/6 were in CR at transplant. In terms of outcome post alloHSCT, two patients relapsed: one received subsequent Brexu-cel, another patient received BTK-I and donor lymphocyte infusion (DLI), both remained alive. Two non-relapsed mortality (NRM) occurred (1 pneumonia, 1 graft failure). Two patients were alive and relapse-free with follow up duration of 4.3 and 9.5 years. Table 2. Figure 2.

**Table 2:** Characteristics and Outcomes of Patients underwent AlloHSCT

	Patient 1	Patient 2	Patient 3	Patient 4	Patient 5	Patient 6
Age/Gender	52/M	57/M	62/F	68/M	43/F	50/F
Prior ASCT	Yes	Yes	No	Yes	No	No
Disease status at transplant	CR	CR	CR	CR	CR	Unknown
Donor type	MUD	MSD	MUD	MSD	MSD	MSD
Conditioning regimen	RIC	RIC	RIC	RIC	RIC	RIC
GVHD Prophylaxis	ATG-CSA-MTX	CSA-MTX	ATG-CSA-MMF	Tacrolimus-MMF	CSA-MMF	CSA-MTX
Progression	No	No	Yes and received Brexu-cel afterwards	No	Yes and received BTK-I and DLI	No
Death	Yes	No	No	Yes	No	No
NRM	Yes, pneumonia	No	No	Yes, Graft Failure and infection	No	No
Follow up duration without progression/death (day)	NA	3464	NA	NA	NA	1580

alloHSCT, allogeneic haematopoetic stem cell transplant; M, male; F, female; CR, complete response; MUD, matched unrelated donor; MSD, matched sibling donor; RIC, reduced intensity conditioning; ATG, anti-thymocyte globulin; CSA, ciclosporin; MTX, methotrexate; MMF, mycophenolate mofetil, brexu-cel, brexucabtagene autoleucel; BTK-I, bruton tyrosine kinase inhibitor; DLI, donor lymphocyte infusion; NRM, non-relapse mortality; NA, not applicable

## Discussion

To our knowledge, this represents the first real-world Asian experience with brexu-cel in R/R MCL. The high ORR (100%) and CRR (80%) are notable given the heavily pretreated cohort, in which all patients had SD/PD at infusion and were refractory to BTK inhibitors. All four patients with follow-up beyond six months remain in CR. Although it is premature to draw conclusions regarding curative potential, these early results are encouraging.

Our outcomes are comparable with the ZUMA-2 study [6, 7], which reported an ORR of 93% and CRR of 67%, with 57% of patients remaining in remission at a median follow-up of 12.3 months. At four years, 44% of patients remained alive, and the median duration of response among patients achieving CR was 58.7 months. Real-world studies from Wang et al. (*n* = 168) and the Center for International Blood and Marrow Transplant Research (CIBMTR) (*n* = 476) reported ORRs of 90–91%, CRRs of 82%, and encouraging PFS outcomes [8, 22]. Similar efficacy has been reported in European and Canadian cohorts [23–27]. Together with Liso-cel (TRANSCEND NHL 001) and Relma-cel (China), CD19 CAR-T therapy has demonstrated substantial effectiveness in R/R MCL [11, 12]. The characteristics and outcomes of clinical trials and real-world CD19 CAR-T therapy in R/R MCL are summarized in Table 3.

**Table 3:** Summary of clinical trial and real world studies using CD19 CAR-T in R/R MCL

Study	CAR-T Product	*N*	Age (median)	Previous BTK-I (%)	ORR (%)	CRR (%)	PFS	OS	≥grade 3 CRS (%)	≥grade 3 ICANS (%)	Infection (%)	NRM (%)
Wang et al., Zuma-2 [6, 7]	Brexu-cel	68	65 (38–79)	100	91	68	mPFS 25.8 months	mOS 46.6 months	15	31	Grade ≥ 3–32%	3^a^
Wang et al., US CAR-T Consortium [22]	Brexu-cel	168	67 (34–89)	86	90	82	1-yr PFS 59%	1-yr OS 86%	8	32	Infection require antimicrobial 33%	1-yr:9
Ahmed et al., CIBMTR [8]	Brexu-cel	476	67 (34–85)	88	91	82	1-yr PFS 63%	1-yr OS 76%	11	30	Clinical significant – 50%	1-yr:8
Nie et al., Stanford University [9]	Brexu-cel	26	67 (50–82)	92%	96	84	1-yr PFS 64–69%	1-yr OS 90–94%	8	38	N.A	N.A
Iacoboni et al., European Early Access [10]	Brexu-cel	33	67 (47–79)	N.A	91	79	1-yr PFS 51%	1-yr PFS 61%	3	36	N.A	15^b^
O’Reilly et al., UK [23]	Brexu-cel	83	68 (41–78)	BTK-I refractory 30%	87	81	1-yr PFS 62%	1-yr OS 74%	12	22	Severe/life-threatening 30% (first month)	1-yr:15
Herbaux et al., DESCART [24]	Brexu-cel	152	68 (39–83)	N.A	85	72	1-yr PFS 46%	2-yr OS 51%	12	15	Clinical Significant − 26%	11^c^
Puckrin et al., Alberta Canada [25]	Brexu-cel	10	Intended (*n* = 15) 63 (31–77)	100	100	70	1-yr PFS 46%	1-yr OS 51%	N.A	N.A	N.A	N.A
Simon et al., Germany and Switzerland [26]	Brexu-cel	85	67 (49–84)	100	88	61	mEFS 25 months	mOS 41 months	13	15	All grade − 31%	1-yr:13
Santoro et al., EBMT [27]	Brexu-cel	233	75 (73–77)	62	91	78	1-yr PFS 62%	1-yr OS 74%	9	22	N.A	1-yr:13
Wang et al., Transcend NHL 001 [11]	Liso-cel	88	69 (36–86)	94	87	74	1-yr PFS 53%	1-yr OS 62%	1	9	Grade ≥ 3–15%	19^d^
Xie et al., China [12]	Relma-cel	59	59 (34–75)	100	71	59	mPFS 16 months	mOS 20 months	7	7	Grade ≥ 3–34%	1-yr:15

BTK-I, bruton tyrosine kinase inhibitor; ORR, overall response rate; CRR, complete response rate; PFS, progression-free survival; OS, overall survival; CRS, cytokine release syndrome; ICANS, immune-effector cell associated neurotoxicity syndrome; NRM, non-relapse mortality; mPFS, median progression-free survival; mOS, median overall survival

^a^ median follow-up of 12.3months

^b^ median follow-up of 10.1months

^c^ median follow-up of 14.2months

^d^ median follow-up of 23.5months

We observed manageable toxicities despite the absence of prophylactic dexamethasone and an elderly cohort, with three of five patients aged ≥ 75 years. No grade ≥ 3 CRS occurred, and only one patient (20%) developed grade 1 ICANS. This compares favourably with ZUMA-2, where grade ≥ 3 CRS and ICANS occurred in 15% and 31% of patients, respectively. Similar rates of severe neurotoxicity and non-relapse mortality have been reported in US consortium, CIBMTR, and EBMT studies [8, 22, 27]. Based on the TRANSCEND study, lisocabtagene maraleucel (liso-cel) may have a more favourable toxicity profile than brexu-cel, although it is not yet available in many Asia-Pacific countries, including Singapore [11]. 

Compared with ZUMA-2 and other real-world cohorts treated between 2016 and 2024, our patients were treated in 2025. Increased institutional experience with CAR-T may have facilitated earlier recognition and management of CRS and ICANS, preventing progression to severe toxicities. Disease burden is also associated with CAR-T–related toxicities, and effective bridging therapy may reduce ICANS [28]. Unlike ZUMA-2, where bridging therapy was limited, all patients in our cohort received bridging therapy, and three of five achieved stable disease before infusion. This may have contributed to the low incidence of severe CRS and ICANS [29]. 

Although acute toxicity was limited, longer follow-up is needed to monitor delayed toxicities such as cytopenia and infection, major contributors to NRM after CD19 CAR-T therapy [30, 31]. Post–CAR-T survivorship care should include multidisciplinary management of cytopenia, infection, secondary malignancies, cardiotoxicity, fertility, and neurological complications [32]. Geriatric assessment may further optimize outcomes in older adults undergoing CAR-T therapy [33]. Overall, appropriate peri–CAR-T optimization appears to support a favourable risk–benefit profile even in elderly patients.

Before CAR-T availability, our preferred approach for R/R MCL was BTK-i with or without alloHSCT consolidation. Although BTK-i remains an effective second-line option, responses are often not durable. In an Asia-Pacific registry, BTK-i achieved an ORR of 74.9% and CRR of 29.1%, but median PFS was only 9.2 months [34]. A large Chinese study similarly demonstrated high rates of relapse after BTK-i, with salvage therapies achieving relatively low CR rates [35]. These findings underscore the challenges of achieving durable disease control after BTK-i failure.

Consequently, discussions with younger and fitter patients have often focused on allo-HSCT while disease remains controlled. However, outcomes are highly dependent on remission status at transplant. EBMT and British registry studies demonstrated poor outcomes in refractory disease and significant NRM, ranging from 24% to 55% [36, 37]. 

In contrast, Liebers et al. reported superior early survival with brexu-cel compared with allo-HSCT (1-year OS 81% vs. 59%; HR 0.39) and markedly lower NRM (3.6% vs. 21%) [38]. In our allo-HSCT cohort, despite most patients being transplanted in CR, two relapsed and two experienced NRM. Given the substantially older median age of the brexu-cel cohort (75 years versus 51 years), brexu-cel appears feasible in a broader population, including patients ineligible for allo-HSCT. These observations support consideration of CAR-T therapy after BTK-i progression and before allo-HSCT.

Despite its proven efficacy, CAR-T therapy remains limited by healthcare infrastructure and funding. Significant global disparities in access persist, even among high-income countries [39]. The scarcity of Asian real-world data further highlights the importance of this report. In Singapore, brexu-cel has been approved since August 2024 but remains incompletely reimbursed, creating access barriers and treatment delays. Based on comparisons between brexu-cel and alloHSCT in our local R/R MCL population, our findings support prioritizing funding for brexu-cel. While alloHSCT remains potentially curative, it carries substantial costs and toxicity-related resource utilization. Improved access to brexu-cel may therefore represent a more practical and beneficial strategy.

Several limitations warrant acknowledgment. The small cohort size precludes definitive comparative analyses and conclusions regarding optimal treatment sequencing. Nevertheless, institutional experiences remain valuable in rare diseases where randomized data are unlikely. Further regional studies are needed to better define survival outcomes and the optimal positioning of cellular therapies in R/R MCL. Our findings contribute to the growing evidence supporting immune-based approaches and the evolving role of cellular therapy in this disease.

## Conclusion

In this report, we detailed an Asian experience utilizing brexu-cel for R/R MCL while contextualizing its use alongside alloHSCT. Although limited by sample size, our findings demonstrate the feasibility of delivering CAR-T therapy within a tertiary Asian centre and support its promising clinical activity in a real-world setting. Notably, meaningful responses in our cohort were observed even among patients with progressive disease at the time of treatment consideration, reinforcing the capacity of CAR-T therapy to induce remission in biologically aggressive disease.

## Data Availability

No datasets were generated or analysed during the current study.
